# The intracoronary wires hand-in-hand technique for uncrossable bilateral microcatheters in CTO lesions: a single-center case series

**DOI:** 10.3389/fcvm.2025.1640101

**Published:** 2025-10-10

**Authors:** Wang Huan, Chen Genrui, Chen Youhu, Lei Xiaolin, Han Peng, Zhang Yamin, Yang Li, Lian Kun, Li Chengxiang, Gao Haokao

**Affiliations:** ^1^ Department of Cardiology, The First Affiliated Hospital of Air Force Military Medical University, Xi’an, China; ^2^ Department of Cardiology, The First Affiliated Hospital of Xi’an Jiaotong University, Xi’an, China

**Keywords:** chronic total occlusion (CTO), percutaneous coronary intervention (PCI), tip-in, rendezvous, major adverse cardiovascular (MACE)

## Abstract

**Background:**

The tip-in and rendezvous techniques are alternative strategies for antegrade conversion when the retrograde microcatheter (MC) cannot cross the chronic total occlusion (CTO) lesion. However, subsequent antegrade MC failure to cross the CTO lesion may increase the failure rate of the CTO procedure.

**Objectives:**

We sought to evaluate the efficacy of the intracoronary wires hand-in-hand (WHIH) technique in this scenario for achieving complete antegrade CTO recanalization.

**Method:**

From September 2023 to December 2024, 14 CTO patients were applied the WHIH technique. The main process of the WHIH technique involves keeping the antegrade and retrograde MCs in close proximity along the retrograde wire, then advancing both wires forward and backward in a hand-in-hand manner along the path created by the retrograde wire until the antegrade wire crosses the CTO lesion. Device success was defined as the achievement of antegrade wire crossing the CTO into the distal vessel after the WHIH technique.

**Results:**

The WHIH success was achieved in all cases. The mean age of the patients was 61.2 ± 12.4 years, and 85.7% of patients were male. The median CTO lesion length was 27.6 mm (range: 7.1–87.3 mm), and the mean J-CTO score was 2.5 ± 0.9. The retrograde approach was predetermined as the first choice in six cases (42.9%), and in eight cases (57.1%) was promptly adopted after the initial antegrade approach failed. Eight cases (57.1%) were accessed through septal collaterals, whereas the remaining six cases (42.9%) via epicardial channels and four of them used ipsilateral epicardial channels. All patients were treated with the tip-in technique, and the median length between two MCs was 4.5 mm (range: 2–20 mm). The WHIH success was achieved in all cases. In-hospital major adverse cardiovascular (MACE) events were not observed.

**Conclusion:**

This intracoronary wires hand-in-hand technique safely and effectively enables antegrade conversion from a retrograde approach, which may serve as a last-resort technique for antegrade access.

## Introduction

The introduction of retrograde techniques in percutaneous coronary intervention (PCI) for chronic total occlusion (CTO) has substantially enhanced the success rates (1). After the retrograde wire crosses the CTO lesion into the antegrade guiding catheter (GC), a microcatheter (MC) is advanced to facilitate the subsequent 300 cm retrograde wire externalization (RWE), such as RG3 (2). The tip-in and rendezvous techniques in antegrade GC are the two main options for achieving antegrade conversion (3–5). The two techniques not only avoid RWE use, thereby reducing potential damage to collateral channels and the ostium of the donor artery, but also potentially lead to a reduction in procedure time and contrast consumption. However, encountering an uncrossable dilemma in bilateral MCs was not uncommon during retrograde CTO-PCI due to complex anatomical features, such as proximal calcification or severe tortuosity. Some related variant strategies have been developed, and all of these collectively form a well-defined category known as “portal techniques” (6).

In this study, we detailed the intracoronary antegrade and retrograde wires hand-in-hand (WHIH) technique, which was successfully used to achieve CTO recanalization in cases where bilateral MCs were uncrossable. This study primarily focused on assessing the efficacy of this technique and aimed to further enrich the “portal techniques”.

## Materials and methods

### Patient populations

From September 2023 to December 2024, at the Xijing Hospital of Air Force Military Medical University, we enrolled 14 patients and performed the WHIH technique due to the failure of both retrograde and antegrade MCs crossing of the CTO lesion after the retrograde wire entered the antegrade GC. The indication for CTO-PCI was the presence of symptomatic angina or extensive silent myocardial ischemia with evidence of viable myocardium in the territory of the occluded vessel, as estimated by echocardiography, SPECT, or cardiac magnetic resonance imaging (MRI). The Ethics Committee of Xijing Hospital approved this study, which was conducted in accordance with the principles of the Declaration of Helsinki, and written informed consents were obtained from all patients. The medical records and coronary angiograms of these patients were reviewed to elucidate the application, effectiveness, and complications of the WHIH technique.

### Intracoronary wires hand-in-hand technique (WHIH) description

The indication of WHIH technique: When the retrograde MC fails to cross the CTO lesion following the retrograde guidewire into the antegrade GC, and the antegrade MC also fails to cross the CTO lesion along the retrograde guidewire.

**Figure 1:**
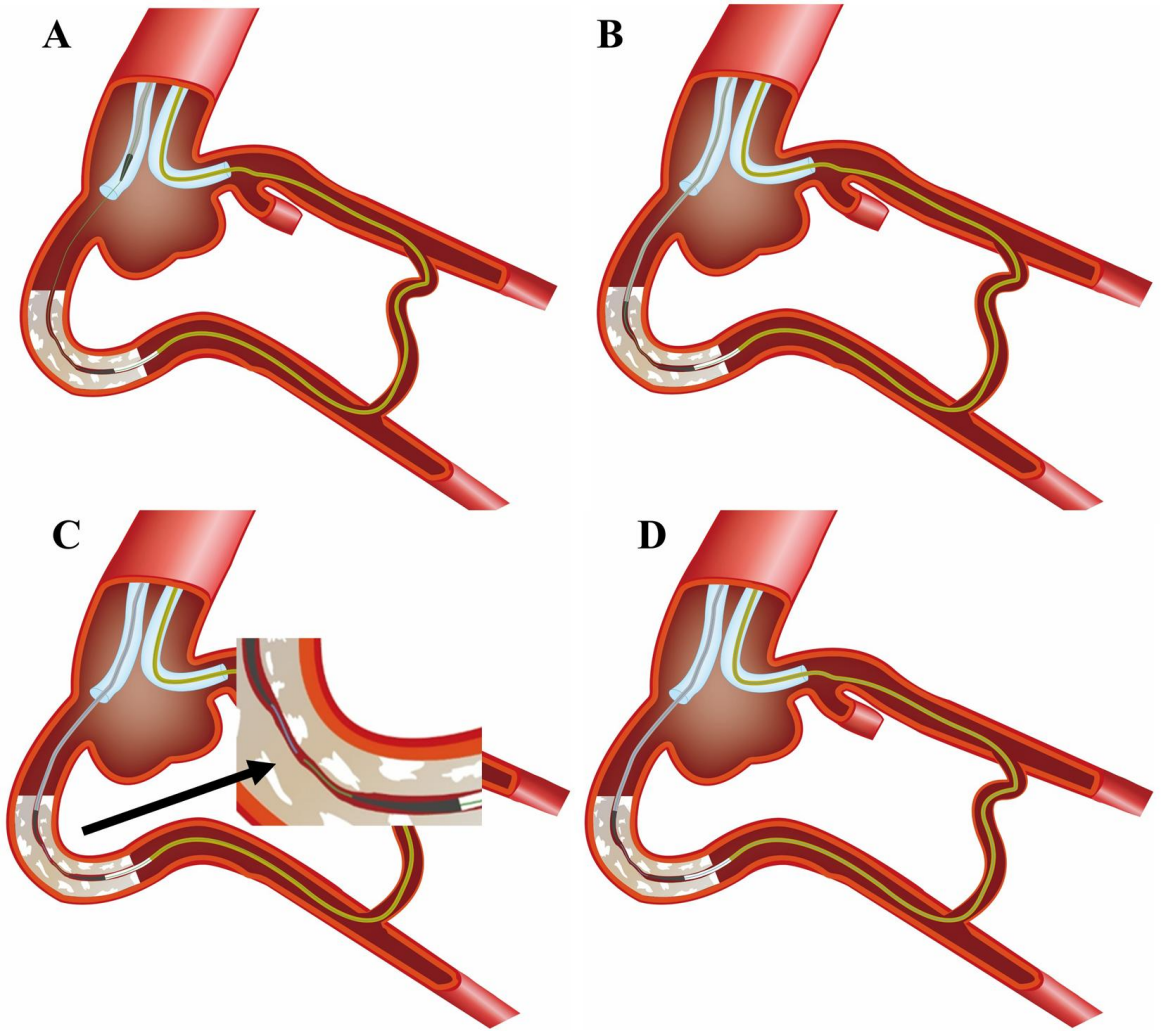
The schematic diagram of the WHIH technique. **(A)** Retrograde wire (green) entered into antegrade GC, but retrograde MC failed to cross the CTO lesion. The tip-in technique used for retrograde wire into antegrade MC. **(B)** Antegrade MC advancement along retrograde wire, but fail to cross the CTO lesion. **(C)** Antegrade workhorse wire (blue) tracks along the path left by the retrograde guidewire during retrieval. **(D)** Prompting the antegrade guidewire (blue) to successfully enter the retrograde MC.

Technical Description (Figure 1): When the retrograde MC fails to cross the CTO lesion following the retrograde guidewire into the antegrade GC, then performing the tip-in technique for retrograde wire into antegrade MC and pushing the retrograde wire as deeply as possible into the antegrade MC to provide strong support (Figure 1A). Subsequently, to maximize the extent of bilateral MCs' penetration into the CTO body, thereby making both MCs as close as possible (Figure 1B). Furthermore, the next most important step is to slowly pull the retrograde guidewire while simultaneously pushing the antegrade guidewire, allowing the two wires to move in a hand-in-hand manner as two operators manipulate them (Figure 1C). When the antegrade wire reaches the retrograde MC tip, manipulate the wire into the retrograde MC as in the Rendezvous technique until reaching the distal vessel (Figure 1D). For this WHIH technique, the same devices as for classic rendezvous or tip-in is required.

### Definitions

A CTO lesion was defined as a complete obstruction of a native coronary artery with Thrombolysis in Myocardial Infarction (TIMI) flow grade 0 and an estimated duration of at least 3 months. The duration of the occlusion was determined by the interval from the last episode of an acute coronary syndrome that was consistent with the location of the occlusion or proved by previous coronary angiography. Heavy calcification was defined as radiopacity noted at the site of the target lesion before contrast injection generally compromising both sides of the arterial lumen. Severe angulation was defined as the vessel body being bent with greater than 45° or exhibiting marked tortuosity. Device success was defined as the antegrade wire crossing the CTO lesion after performing the WHIH technique. Technical success was defined as a visual assessment of recovery antegrade TIMI flow grade III in the target vessel and residual stenosis < 30%. In-hospital MACEs included cardiac death, PCI-related MI, ischemia-driven revascularization PCI, or emergency cardiac surgery.

### Statistical analyses

Continuous parameters were presented as mean ± SD or median (Q1–Q3). Categorical variables were presented as percentages. The Student's *t*-test was used to estimate the differences in continuous variables. A *P* value < 0.05 was chosen to indicate a significant difference. All analyses were performed using SPSS for Windows 13.0.

## Results

### Baseline demographics and angiographic characteristics

A total of 14 patients underwent successful PCI for CTO lesions using this WHIH method. Baseline clinical characteristics are shown in Table 1. The mean age of the patients was 61.2 ± 12.4 years, and 85.7% were male. Almost half of the patients had multiple risk factors, and 71.4% had a history of previous PCI. 92.9% had unstable angina with CTO lesions, and 78.6% had multivessel disease. The rate of occlusion sites was similar in the RCA (42.9%) and the LAD (42.9%). 95.0% of the lesions had moderate to severe calcification, and 71.4% had an occlusion length of ≥20 mm and with a blunt stump or abrupt proximal cap. The median CTO lesion length was 27.6 mm (range: 7.1–87.3 mm). The CTO complexity calculated using J-CTO score, the mean J-CTO score was 2.5 ± 0.9.

**Table 1:** Baseline clinical data and angiographic information.

Variables	WHIH (*n* = 14)
Age, yrs	61.2 ± 12.4
Gender, male *n* (%)	12 (85.7)
Hypertension *n* (%)	11 (78.6)
Diabetes *n* (%)	5 (35.7)
Hyperlipemia *n* (%)	7 (50.0)
Smoker *n* (%)	9 (64.3)
Prior MI *n* (%)	6 (42.9)
Prior PCI *n* (%)	10 (71.4)
Prior CABG *n* (%)	0 (0.0)
Clinical diagnosis	
Stable angina, *n* (%)	1 (7.1)
Unstable angina, *n* (%)	13 (92.9)
LVEF %	50 ± 10
≤35%	3 (21.4)
≥35%	11 (78.6)
Multivessel coronary disease *n* (%)	11 (78.6)
Target CTO vessel *n* (%)	
Proximal LAD	3 (21.4)
Middle LAD	3 (21.4)
Proximal LCx	2 (14.3)
Middle LCx	0 (0.0)
Proximal RCA	3 (21.4)
Middle RCA	3 (21.4)
CTO assessment *n* (%)	
Length > 20 mm	10 (71.4)
Bending > 45°	3 (21.4)
Calcification	7 (50.0)
Blunt stump/abrupt in proximal cap	10 (71.4)
Re-try lesion	4 (28.6)
Diffuse lesion in distal vessel	4 (28.6)
Side branch at landing zone	6 (42.9)
J-CTO score	
Mean J-score	2.5 ± 0.9
≥3 *n* (%)	8 (57.1)

Results are expressed as the mean ± SD.

WHIH, wires hand-in hand; MI, myocardial infarction; PCI, percutaneous coronary intervention; CABG, coronary artery bypass graft; LVEF, left ventricular ejection fraction; CTO, chronic total occlusion; LAD, left anterior descending artery; LCX, left circumflex artery; RCA, right coronary artery; MI, myocardial infarction, PCI, percutaneous coronary intervention; CABG, coronary artery bypass graft; LVEF, left ventricular ejection fraction.

### Procedure characteristics and outcomes

The procedural characteristics were listed in Table 2. All procedures were finished with 7 F GC. 71.4% of the patients were with bilateral GC usage. The retrograde approach was pre-determined as the first choice in six cases, and promptly adopted after the first antegrade approach failed in eight cases. Eight cases (57.1%) were accessed through septal collaterals, whereas the remaining six cases (42.9%) via epicardial channels, four of which were ipsilateral epicardial channels. 57.1% of cases with the reverse controlled antegrade and retrograde subintimal tracking (r-CART) technique and all patients achieved retrograde wire across CTO lesion into antegrade GC with stiffer wires. The tip-in technique was successfully performed to advance the retrograde wire into the antegrade MC in all patients. The median distance between antegrade and retrograde MCs was 4.5 (rang: 2–20) mm. Meanwhile, in all cases, the WHIH technique for antegrade workhorse wires such as the Sion (Asahi Intecc) or Sion Black (Asahi Intecc) was attempted, and the device success rate was 100%. There was one case where, after successfully completing the WHIH technique, the antegrade system could not cross the CTO lesion despite trying multiple methods. Eventually, the retrograde wire was repeated to enter another subintimal path, achieving antegrade conversion. There were 3 cases of rotational atherectomy (ROTA) usage due to severely calcified lesions. The technical success rate was 100.0%. No in-hospital MACEs or procedural complications occurred.

**Table 2:** Procedural characteristics of the study population.

Variables	WHIH (*n* = 14)
GC usage *n* (%)	
Bilateral GC	10 (71.4)
Unilateral GC	4 (28.6)
Ping-pang GC	3 (21.4)
Single GC	1 (7.1)
Retrograde approach *n* (%)	
Primary	6 (42.9)
Promptly after failed antegrade	8 (57.1)
Retrograde collaterals used *n* (%)	
Septal channel	8 (57.1)
Epicardial channel	6 (42.9)
Isplateral epicardial channel	4 (66.7)
Retrograde wires into antegrade GC *n* (%)	
Pilot wires	8 (57.1)
Gaia wires	2 (14.3)
UB3 wire	4 (28.6)
Retrograde MCs uncross the CTO lesion *n* (%)	14 (100.0)
Types of retrograde MCs *n* (%)	
Finecross MCs	2 (14.3)
Corsair MCs	9 (64.3)
RAD-pass MCs	3 (21.4)
Tip-in method usage	14 (85.7)
Antegrade Corsair MCs uncross CTO lesion	14 (100)
Antegrade Wires used for hand-in hand	
Workhorse wire (sion/sion black)	14 (100%)
Success of Wire hand-in hand Technique (WHHT)	14 (100.0)
Distance between antegrade and retrograde MCs (mm)	
Median distance (mm)	4.5 (rang 2–20)
<5 mm *n* (%)	6 (42.9)
≥5 mm *n* (%)	8 (57.1)
ROTA Usage *n* (%)	3 (21.4)
Fluoroscopy time, min	141.5 ± 66.3
Procedure time, min	243.5 ± 69.4
Contrast volume, ml	296.8 ± 73.7

WHIH, wires hand-in hand; CART, controlled antegrade retrograde subintimal tracking; TIMI, thrombolysis in myocardial infarction; MC, microcatheter; ROTA, rotational atherectomy; NA, not applicable.

The outcomes, such as success rates and complication rates, between WHIH and “portal techniques” (References 4 and 5) showed no significant difference. However, procedural parameters revealed a statistically significant difference in procedure time (*p* = 0.022) (see Supplementary Table S1).

### Two representative cases of WHIH practice

**Figure 2:**
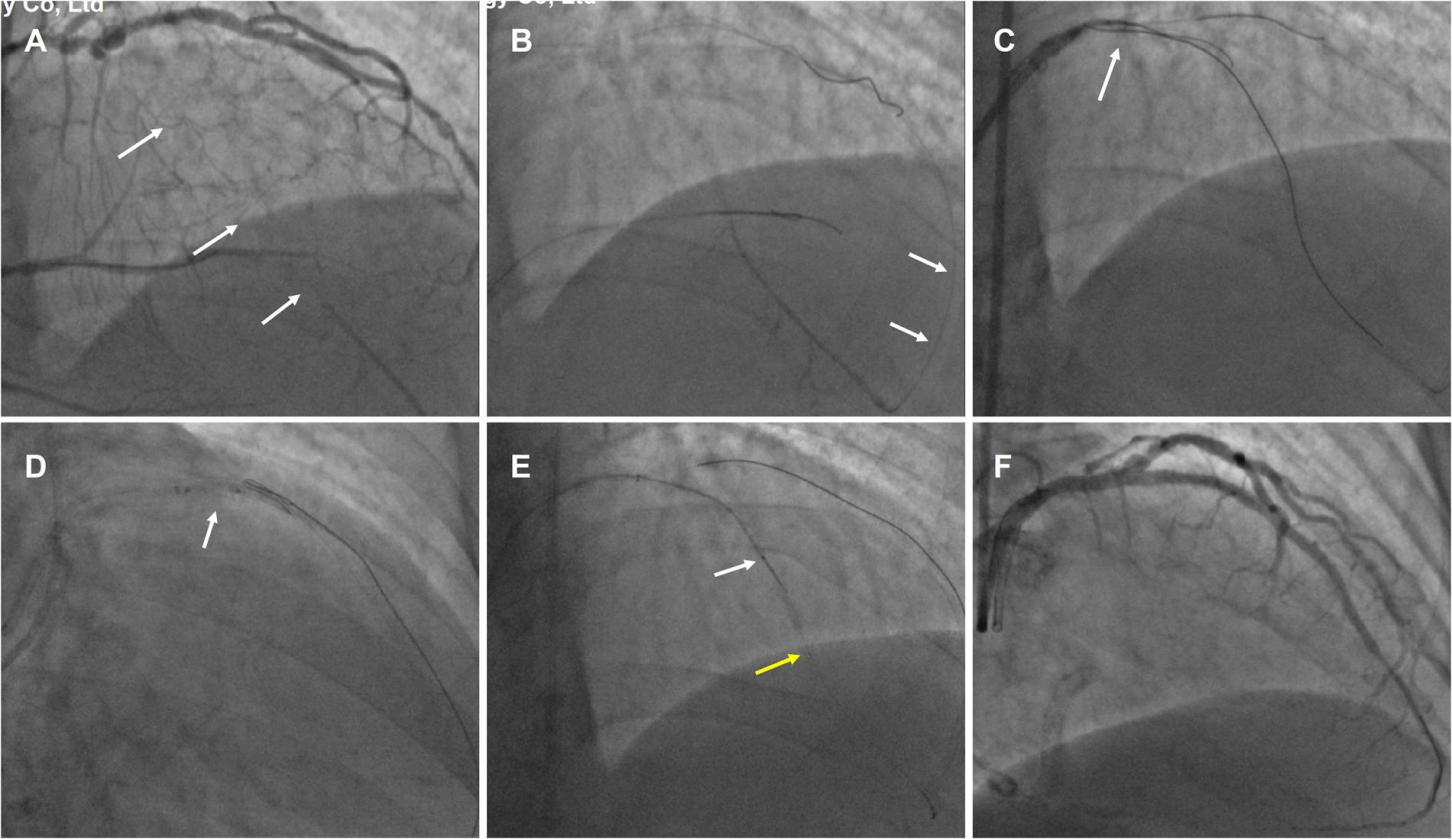
An example of the WHTH between two MCs over a long distance. **(A)** mid-LAD CTO (white arrow) after the big first diagonal (D1) vessel and with ambiguous entry. **(B)** Retrograde Suoh 03 wire was advanced through the ipsilateral CC (white arrow) into the distal LAD. **(C)** the retrograde UB3 wire (white arrow) crossed the CTO segment but could not enter the antegrade GC. **(D)** After performing the reverse CART technique (white arrow), the retrograde UB3 wire into the antegrade extension catheter. **(E)** WHIH technique preformed when antegrade MC (white arrow) and retrograde MC (yellow arrow) uncrossable CTO lesion. **(F)** Excellent angiographic results were achieved after the DESs implantation.

1) An example of the WHTH between two MCs over a long distance in Figure 2.

A 69-year-old male was readmitted for secondary PCI for LAD-CTO with significant anterior wall ischemia on SPECT tests. Diagnostic CAG showed the mid-LAD CTO after giving off the big first diagonal (D1) vessel, and with ambiguous entry, and distal LAD flow filled by apical epicardial collateral channels (CCs) from the proximal diagonal branch vessels (Figure 2A, Supplementary Video S1**)**. IVUS indicated diffuse calcification with aneurysmal dilation in the proximal segment of LAD, and the CTO entrance was unclear. Therefore, retrograde approach was first attempted; Suoh 03 (Asahi Intecc) wire was advanced over 150 mm Finecross MC (Terumo Corporation) MC through the ipsilateral CC into the distal LAD (Figure 2B). Then the UB3 wire was manipulated into the CTO lesion up to the proximal CTO cap. Due to the angulation, the wire could not enter the antegrade GC (Figure 2C), and the retrograde MC was also unable to cross the CTO lesion. Subsequently, the antegrade Gaia 3 wire within the Corsair catheter (Asahi Intecc) was inserted into the supposed proximal cap but deviated from the retrograde wire. After performing the reverse CART technique, the retrograde UB3 wire was manipulated into the antegrade extension catheter (Figure 2D). However, the retrograde MC was still unable to cross the CTO segment even with conventional balloon anchoring in the antegrade GC. Then, the tip-in technique was performed successfully. Unexpectedly, the antegrade MC advancement over the retrograde wire failed to cross the CTO lesion, and there was still approximately a 15 mm gap between the two MCs even when they were positioned as closely as possible. Subsequently, the antegrade guidewire was pushed smoothly while the retrograde guidewire was pulled simultaneously in a hand in hand manner until the antegrade wire entered the retrograde MC (Figure 2E, Supplementary Video S2). The IVUS confirmed that the wire was located in the subintimal space beyond the D1 vessel and revealed evidence of severe localized calcification in the LAD CTO. Excellent angiographic results were achieved after the drug eluting stens (DESs) implantation (Figure 2F).

**Figure 3:**
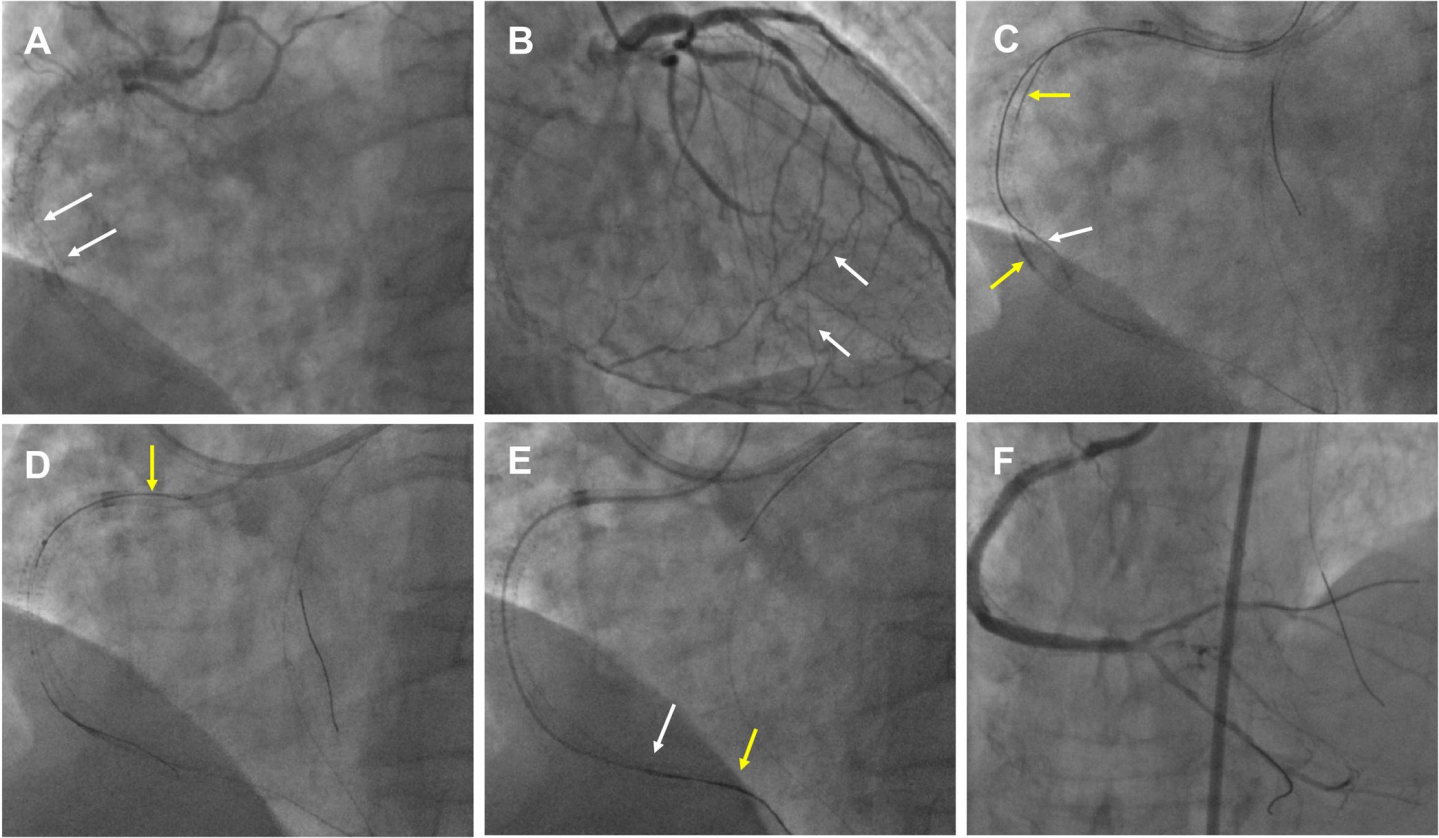
An example of the WHIH in an in-stent high-resistance occlusion lesion. **(A)** RCA in-stent re-occlusion (white arrow) with deformed middle stents that had collapsed to one side. **(B)** RCA distal bifurcation vessels filled by contralateral septal vessels (white arrow). **(C)** Antegrade wire (white arrow) and retrograde wire (yellow arrow) were entered the RCA middle segment. **(D)** The tip-in technique performed for antegrade MC advancement over a retrograde wire (yellow arrow). **(E)** WHIH technique preformed when antegrade MC (white arrow) and retrograde MC (yellow arrow) uncrossable CTO lesion. **(F)** Excellent angiographic results were achieved after the DESs and DCB.

2) An example of the WHIH technique in a high-resistance in-stent occlusion lesion in Figure 3

A 65-year-old man with a 10-year history of hemodialysis for chronic kidney failure has severe angina symptoms. Five months ago, DESs were implanted in the LAD for severe stenosis, and the in-stent CTO in the RCA was also treated. However, during the RCA CTO-PCI, the wires were positioned outside the stents in the middle segment, and the stents were dilated using 3.0/3.5 mm non-compliant balloons, achieving TIMI 3 flow in the posterior descending (PD) and posterolateral (PL) vessels. For the second PCI this time, dual CAG showed RCA in-stent re-occlusion from the proximal to just before the distal bifurcation, which was filled by contralateral vessels and featured deformed stents that had collapsed to one side (Figures 3A,B, Supplementary Video S3). Antegrade approach was first attempted, but the wires only extended into the mid-segment of the RCA and were located outside the deformed stents. Next, the retrograde Suoh3 wire crossed the septal vessel into PD vessels with Corsair MC 150. After several attempts with stiffer wires to penetrate the CTO failed, a knuckled Fielder XT-A (Asahi Intecc) wire was successfully advanced into the RCA middle segment. However, the retrograde Corsair MC was hindered at the end of the distal stent. Antegrade Fielder XT-A knuckled wire was pushed into the RCA middle segment, and after 1.5/2.0 mm balloon dilation, IVUS showed that the retrograde wire was located within the in-stent structure (Figure 3C). Subsequently, the retrograde Corsair MC was replaced by TURNPIKE150 MC, which was advanced to the RCA middle segment. After the reverse-CART technique, a new Pilot 200 wire was retrogradely manipulated into the antegrade GC (Supplementary Video S4). However, the TURNPIKE150 MC only reached the RCA 2 segment. The tip-in technique was performed to facilitate antegrade MC advancement over the retrograde wire (Figure 3D, Supplementary Video S5). Due to stent higher resistance, the antegrade MC only extended to the RCA distal stent segment, and in this dilemma, the WHIH technique was performed, and the antegrade wire was smoothly advanced into the ostium of the PD vessel while tracing along with the retrograde wire (Figure 3E, Supplementary Video S6). The final angiography showed good results with DESs and DCB (Figure 3F).

## Discussion

To our knowledge, the present study is the first report to demonstrate a series of successful cases using the intracoronary WHIH technique to achieve antegrade conversion during retrograde CTO recanalization. This technique serves as a practical and promising alternative in scenarios where both antegrade and retrograde MCs fail to cross the CTO lesion.

Allana SS et al. (7) reported that the tip-in and rendezvous techniques were utilized in 3.0% of procedures, and the most common reasons for the use of these techniques were inability of the retrograde MC to reach the antegrade GC (35.4%), operator preference (18.8%), and use of epicardial collaterals (16.7%) in the PROGRESS-CTO study. CTO-PCI with ipsilateral collateral channels is challenging during retrograde CTO PCI. Azzalini L et al. (2) reported the use of the tip-in technique in 9% of ipsilateral retrograde procedures and the Rendezvous technique in 7% of ipsilateral cases. During the retrograde approach, the tip-in and rendezvous techniques, which were mostly performed in antegrade GC, were used at a high rate based on operator preference and clinical scenarios in our center. In this study, all the retrograde MC uncross the CTO and 28.6% cases with the use of ipsilateral collateral channels; therefore, the tip-in technique was applied in all cases. At the subsequent pivotal step, where the antegrade MC crossing of the CTO lesion along the retrograde wire was unfeasible due to high calcification, severe angulation, or high resistance in the stent struts, we performed the WHIH technique to achieve antegrade conversion. Due to the complex CTO, thereby increasing the procedural attempt time with the WHIH technique.

When facing the challenge of bilateral MCs' inability to pass through the CTO lesion during the retrograde CTO procedure, several variants and solutions (8–11) are introduced to solve this issue, which is called the “portal technique" (6). This WHIH technique shares the same concept as the “Wire Rendezvous and Chasing Wire Technique” reported by Nakabayashi K et al. (10). He pointed out that a very long CTO or a CTO requiring the CART technique might pose risks when using this chasing wire technique. In a similar setting, Wu EB (11) also suggested advancing an antegrade rotablator wire into the retrograde MC if there was a short remaining distance between the bilateral MCs. But in our series of cases, the CTO length, CART/reverse-CART usage, and the remaining distance between the two MCs were not pivotal factors. The underlying mechanism of this technological success was that the channel created by the retrograde wire formed a “stable tunnel”, even though the dissection spaces inside it. The existence of calcified plaque allowed the antegrade workhorse wires to cross the CTO lesion more effectively and alleviated concerns about acute re-occlusion when the retrograde wire retrieved.

The WHIH technique offers potential advantages, including simplifying the retrograde approach without extra device usage. Thus, based on our single-center experience, the WHIH technique could serve as a last-resort solution in scenarios where both antegrade and retrograde microcatheters fail to successfully cross the CTO lesion.

## Limitations

This study has several limitations. First, the sample size in this single center is small, which may result in sampling bias. Second, while the implementation of the WHIH procedure in the complex CTO cohort shows a higher success rate, it is imperative to consider that the observed success may be attributable to the considerable expertise, discerning case selection, and strategies adopted by highly experienced operators. Therefore, caution should be exercised regarding the generalizability of the WHIH technique to other centers. Third, due to the retrospective design, some causal inferences cannot be made. These limitations highlight the necessity and importance of launching a prospective study with more cases and a randomized controlled design in the future.

## Conclusions

This intracoronary wires hand-in-hand technique safely and more effectively accomplishes the antegrade conversion without adding extra devices, which could serves as a last-resort solution in cases where both antegrade and retrograde microcatheters fail to successfully cross the CTO lesion.

## Data Availability

The raw data supporting the conclusions of this article will be made available by the authors, without undue reservation.
