# Supplementary material for: The intracoronary wires hand-in-hand technique for uncrossable bilateral microcatheters in CTO lesions: a single-center case series
Source: Front Cardiovasc Med. 2025 Oct 10;12:1640101. doi: 10.3389/fcvm.2025.1640101 (PMC12549595; doi:10.3389/fcvm.2025.1640101)
Supplement: Supplementary file 7 [file Table1.docx]

**Supplement Table 1. The procedural parameters in WHIH and two rendezvous studies (Ref 4 and 5)**

|  | **WHIH (n=14)** | **Re 4. Rendezvous (n=20)** | **P value** |
| --- | --- | --- | --- |
| Fluoroscopic time (min) | 141.5±66.3 | 131.3±138.2 | 0.78 |
| Procedure time (min) | 243.5±69.4 | 176.7±92.5 | 0.02 |
| Contrast volume (ml) | 296.8±73.7 | 320.0±65.3 | 0.35 |
|  |  |  |  |
|  | **WHIH (n=14)** | **Re 5. Intracoronary rendezvous (n=10)** | **P value** |
| Fluoroscopic time (min) | 141.5±66.3 | 124.5±63.7 | 0.53 |
| Procedure time (min) | 243.5±69.4 | 197.0±88.6 | 0.18 |
| Contrast volume (ml) | 296.8±73.7 | 234.5±78.0 | 0.11 |

WHIH: Wires hand-in hand; Ref 4/5: Reference 4/5.
